# Preparation and fabrication of a full‐scale, sagittal‐sliced, 3D‐printed, patient‐specific radiotherapy phantom

**DOI:** 10.1002/acm2.12162

**Published:** 2017-08-30

**Authors:** Daniel F. Craft, Rebecca M. Howell

**Affiliations:** ^1^ Department of Radiation Physics The University of Texas MD Anderson Cancer Center Houston TX USA; ^2^ Medical Physics Program The University of Texas Graduate School of Biomedical Sciences at Houston Houston TX USA

**Keywords:** 3D printing, phantoms

## Abstract

**Purpose:**

Patient‐specific 3D‐printed phantoms have many potential applications, both research and clinical. However, they have been limited in size and complexity because of the small size of most commercially available 3D printers as well as material warping concerns. We aimed to overcome these limitations by developing and testing an effective 3D printing workflow to fabricate a large patient‐specific radiotherapy phantom with minimal warping errors. In doing so, we produced a full‐scale phantom of a real postmastectomy patient.

**Methods:**

We converted a patient's clinical CT DICOM data into a 3D model and then sliced the model into eleven 2.5‐cm‐thick sagittal slices. The slices were printed with a readily available thermoplastic material representing all body tissues at 100% infill, but with air cavities left open. Each slice was printed on an inexpensive and commercially available 3D printer. Once the printing was completed, the slices were placed together for imaging and verification. The original patient CT scan and the assembled phantom CT scan were registered together to assess overall accuracy.

**Results:**

The materials for the completed phantom cost $524. The printed phantom agreed well with both its design and the actual patient. Individual slices differed from their designs by approximately 2%. Registered CT images of the assembled phantom and original patient showed excellent agreement.

**Conclusions:**

Three‐dimensional printing the patient‐specific phantom in sagittal slices allowed a large phantom to be fabricated with high accuracy. Our results demonstrate that our 3D printing workflow can be used to make large, accurate, patient‐specific phantoms at 100% infill with minimal material warping error.

## 1 INTRODUCTION

In radiation therapy, commercially available anthropomorphic phantoms can be used for end‐to‐end quality assurance (QA) of new treatment techniques. Such phantoms are generally available in only four forms: male, female, child, and infant. These phantoms have average body mass indices, but most patients' individual anatomy differs greatly from that of the representative phantoms. For example, a postmastectomy woman with a high body mass index is not accurately represented by the standard adult female phantom. A patient‐specific phantom that accurately represents an individual's specific anatomy has greater validity than the standard phantom as a model in a variety of research and clinical applications. Patient‐specific phantoms have many potential uses in radiotherapy but are generally not commercially available. This lack can be attributed to the development time and expense required to individualize the production process. Three‐dimensional (3D) printing is one tool that potentially can be used to inexpensively custom‐fabricate patient‐specific phantoms.

Several studies published in recent years have shown the advantages of 3D‐printed phantoms for use in radiotherapy and in other areas of medicine. Ehler et al.^1^ printed a shell of a generalized head and filled it with wax to be used for intensity‐modulated radiation therapy QA. Ger et al.^2^ also printed a shell of a head, but filled it with variable‐density materials to make a heterogeneous head phantom. Similarly, Gear et al.^3^ printed liquid‐fillable shells, but of patient‐specific organs, to be used as molecular imaging phantoms. Nattagh et al.^4^ created a training phantom for ultrasound‐guided needle insertion and suturing during gynecologic brachytherapy procedures. As proposed by Burleson et al.,^5^ 3D‐printed exterior molds of a patient can be useful as a way to fit electron bolus before treatment for patients with open wounds or sensitive skin. 3D‐printed patient‐specific phantoms have also been found to be helpful in surgical planning^6^ and in the education of medical residents for surgeries of the liver^7^ and brain.^8^ In general, patient‐specific phantoms could be used not only for end‐to‐end QA of new radiation therapy techniques but also to perform QA for routine treatments on patients with highly atypical anatomy.^9^

Despite the conceptual simplicity of 3D‐printed patient‐specific phantoms, they have several key limitations. Two of these are the small size of most commercially available 3D printers^10^ and the tendency of printed objects to warp or become distorted while printing.^5^ Warping happens because of the way objects are printed in successive layers. Different layers cool and contract at different rates, causing the object being printed to curl upward from the build platform as layers separate from each other. In severe cases, the warping object can impede the path of the extruder and cause the model to stop printing. In other cases, this warping is minor, affecting only the bottom few layers. The warping effect is heightened when large surfaces of the object being printed are in contact with the print bed. Warping is also much more problematic when an object is being printed at 100% infill (solid) rather than at a lower infill percentage or as a hollow shell. Warping is most prevalent with 3D printers that use fused deposition modeling technology, but other printing technologies, like stereolithography, are generally even more limited in total print volumes making them impossible to use for large phantoms.

Many of the published studies have minimized the impact of warping by printing only a shell of the phantom and then filling it with water or wax. These liquid‐filled phantoms are more complicated to design, however, and are homogenous without internal air gaps. In addition, all of the reported studies of 3D‐printed phantoms are for small anatomical regions (i.e., head or smaller), where the impact of warping is minimal. The goal of this study was to develop and test a 3D printing workflow with minimal warping error that can be used to print any large anatomical region with 100% infill representing tissue, while allowing for inclusion of low‐density air‐filled regions.

## 2 MATERIALS AND METHODS

**Figure 1:**
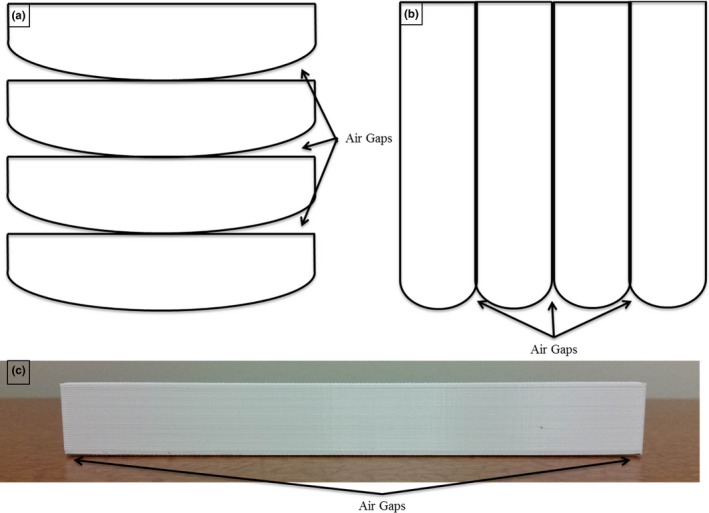
Slice orientation and material warping. This is a representation of what a square phantom might look like if printed in different orientations. (a) and (b) show how warping could affect slices printed in the axial and sagittal planes, respectively, due to the edges curling up from the print bed. All parts are shown as if they were printed with their inferior aspect in contact with the print bed. Note that in (b), there is overall less warping from the desired square, and the warping that does happen does not affect the contact between slices. (c) Picture of a 3D‐printed block with warping observed on the edges that were in contact with the printing bed during printing.

We printed the phantom as eleven 2.5‐cm‐thick sagittal slices, with the inferior aspect of each slice in contact with the print bed. This approach was chosen with the goal of minimizing the extent and effects of material warping on our phantom. 3D‐printed objects warp primarily from their contact with the print bed, so orienting all the slices in the sagittal plane accomplishes two things. First, only 2.5 cm of the slices makes contact with the print bed, rather than the entire width of the phantom, so there is less overall surface area to warp. Second, because any warping that does occur is localized to the inferior aspect of the slice, it does not obstruct the contact points between slices. The superiority of sagittal slices over axial slices in respect to warping is illustrated in Fig. 1.

### 2.A 3D printing file preparation

**Figure 2:**
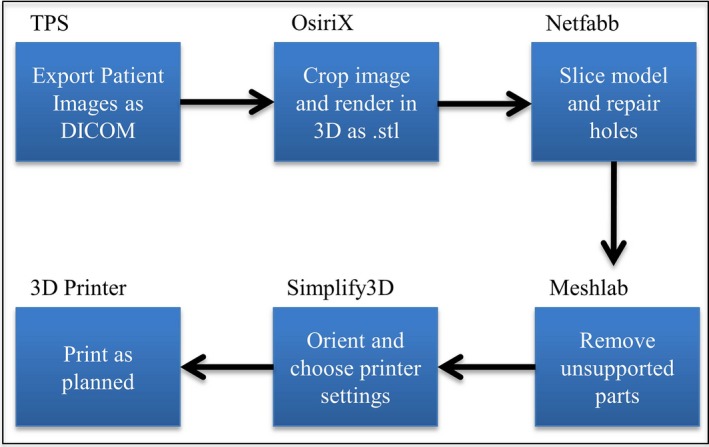
Workflow diagram showing the steps and software used to prepare files for 3D printing, from treatment planning (TPS) to product.

We designed our printed phantom directly from a computed tomography (CT) image of a 76‐year‐old woman who had undergone a left‐sided mastectomy. This patient is part of an institutional review board–approved cohort of patients for retrospective studies. The first step in developing ready‐to‐print files was to export the patient‐of‐interest's DICOM data from the clinical planning system, Pinnacle^3^ (Philips Healthcare; Andover, MA, USA), to the DICOM imaging software OsiriX (Pixmeo; Bernex, Switzerland). The sequence of steps is diagramed in Fig. 2.

Using OsiriX's voxel value reassignment function, we set the Hounsfield unit (HU) value of voxels outside the area of interest to −1024. This effectively cropped those areas from the final 3D skin rendering. We cropped the arms to simplify printing and the head to preserve patient anonymity. Once the CT image was processed within OsiriX, we used the 3D surface rendering function to produce a 3D model of the image for all voxels with an HU above a threshold of −500. That threshold value keeps all soft tissue and bone as one material, but leaves open air cavities such as the trachea and lungs. This whole‐body model was then exported to the 3D model manipulation software netfabb (netfabb GmbH; Parsberg, Germany), now Autodesk (San Rafael, CA, USA).

Netfabb has a special cutting function that we used to slice the original whole‐body model into eleven 2.5‐cm‐thick sagittal slices. Each individual slice was then exported into MeshLab, another 3D model manipulation program. This open source and free to use system developed by the 3D‐CoForm project allows piece‐by‐piece editing and cropping. In MeshLab, each slice was examined for any overhanging or free‐hanging parts that would cause printing errors. For example, sagittal slices that intersect the lungs often left parts of the bronchi unsupported, so those parts were deleted. These sections were generally small. Once every slice was cleared of overhanging parts, they were transferred to Simplify3D (Simplify3D, Cincinnati, OH, USA). This software translates 3D models into g code, the language used by our 3D printer to define print jobs. Each slice was oriented with its flat 2.5‐cm‐thick inferior aspect on the print bed. The print‐ready files were saved onto an SD card.

### 2.B Phantom fabrication

All eleven slices were printed by a Gigabot 2.0 printer (re:3D; Houston, TX). The Gigabot has the capacity to print objects with dimensions up to 60 cm × 60 cm × 54 cm (x, y, z) with a layer resolution of 100–300 microns and x‐y resolution of 4 microns. This build volume is much larger than those used in previous studies.^1^, ^3^, ^11^ Each slice was printed with a 300‐micron layer resolution at 100% infill using 2.85‐mm polylactic acid (PLA) filament (re:3D; Houston, TX, USA). Many different materials' radiological properties have been studied, but the two most widely used are PLA and acrylonitrile butadiene styrene (ABS). Both materials are nearly identical in price and printing speed, and both have similar radiological properties to water.^1^, ^5^, ^10^ However, ABS warps considerably more than PLA which makes it extremely difficult to use for large objects printed at 100% infill.^5^, ^12^ PLA was chosen primarily for its superior warping characteristics.

The settings used by the printer can strongly affect the degree of warping observed, so each of the settings used by this experiment were informed based on previous experience. Many objects were printed with 100% infill using various settings, and the settings below were found to be the best practice for minimizing warping and limiting print failures. The printing bed was set to 60°C and the print nozzle to 225°C. The extrusion multiplier, or flow rate, was set to 90%, and the nozzle's print speed was 60 mm/s. The printing bed is made of BuildTak (Ideal Jacobs Corporation, Maplewood, NJ, USA), but unlike many other experiments with 3D printing, no other tape or adhesive was applied to the bed. As each slice was printed, we recorded the total time to print it, the mass of the material used, and the total cost of the material. Average slice and total phantom printing parameters were then calculated. After the 3D printing was completed, we drilled two holes across each slice so that plastic immobilization rods could be placed to hold the phantom slices together.

### 2.C Material analysis

To determine the attenuation properties of the PLA material used for the phantom, we printed several 5 cm × 5 cm × 5 cm cubes of PLA. The cubes were imaged on a Phillips Brilliance Big Bore CT scanner (Philips Healthcare, Andover, MA, USA). The CT number of each block was recorded, and the blocks were weighed and measured to determine their volume, density, and print accuracy.

We additionally printed larger blocks of PLA that could be used to perform percent depth dose (PDD) measurements. The blocks consisted of one 20 cm × 20 cm × 2.5 cm block, two 20 cm × 5 cm × 2.5 cm blocks, a 20 cm × 2.5 cm × 5 mm strip, and a 20 cm × 2.5 cm × 3 mm strip. Holes were incorporated into the block design to accommodate the Exradin A1SL small volume ion chamber (Standard Imaging Inc., Middleton, WI, USA). By stacking the various blocks in different orders, the ion chamber could be positioned at any depth from 1 to 15 cm at 1 cm increments. By using the 5 and 3 mm strips, smaller measurement increments were possible.

We performed our measurements using a Varian 2100 linear accelerator (Varian Medical Systems, Palo Alto, CA, USA). PDDs were measured for 6‐ and 18‐MV photon energies and 4, 6, 7, 9, 11, 12, 16, and 20‐MeV electrons. At each measurement point, we recorded three measurements with our ion chamber, and for all measurements, the PLA blocks had solid water sheets on either side to ensure scatter equilibrium. All measurements were done with a 100 cm source‐to‐surface distance, and all electron measurements were done using a 10 cm × 10 cm electron applicator.

The dose at each measurement location was calculated by following the protocol in the American Association of Physicists in Medicine Task Group 25 report.^13^ Collisional stopping power ratios were obtained from the NIST program ESTAR.^14^

After measuring the PDDs, we acquired a CT of the PLA PDD blocks in the PDD measurement configuration and imported it into our treatment planning system Pinnacle^3^ V9.10 (Philips Healthcare, Andover, MA, USA). For each measured photon and electron energy, a single‐field treatment plan was created, and the calculated dose was recorded at each measurement location to create a calculated PDD curve. Based on the CT scan of the blocks, the planning system's CT calibration curve estimated PLA's physical density to be 1.09 relative to water, which is not correct. To rectify this, the density was manually overwritten to be 1.20 relative to water in order to accurately calculate dose.

### 2.D Phantom verification

We evaluated the phantom slices both individually and collectively. Before drilling the holes for the placement of the immobilization rods, a CT scan was acquired of each individual slice on a Philips Brilliance Big Bore (Philips Healthcare, Andover, MA, USA). We also measured the thickness of each slice at its superior, anterior, and inferior aspects with submillimeter resolution calipers. We defined the per‐slice printing accuracy in two ways: measured accuracy and volumetric accuracy. The measured accuracy was defined by calculating the average discrepancy between the measured and planned thicknesses at the top, middle, and bottom of each slice. The volumetric accuracy of each phantom slice was defined by comparing the volume of the 3D rendering of the CT scan with the model created for printing.

After the rods were placed, we acquired a CT scan of the entire phantom. The assembled phantom accuracy was evaluated by registering its CT image with the original patient CT image and examining the slice‐by‐slice alignment in all three planes. In addition, average CT numbers were measured throughout the phantom and patient images to determine the CT number discrepancy in various anatomic regions.

## 3 RESULTS

### 3.A 3D printing file preparation

Preparing the eleven slices from the original CT data was straightforward and simple. The process of cropping the CT scan, converting it to a 3D model, and slicing it into 2.5‐cm slices took less than 30 min once we were familiar with the workflow. Preparing each individual slice for printing took slightly longer, approximately 10 min per slice, or 2 hr in total.

### 3.B Phantom fabrication

**Figure 3:**
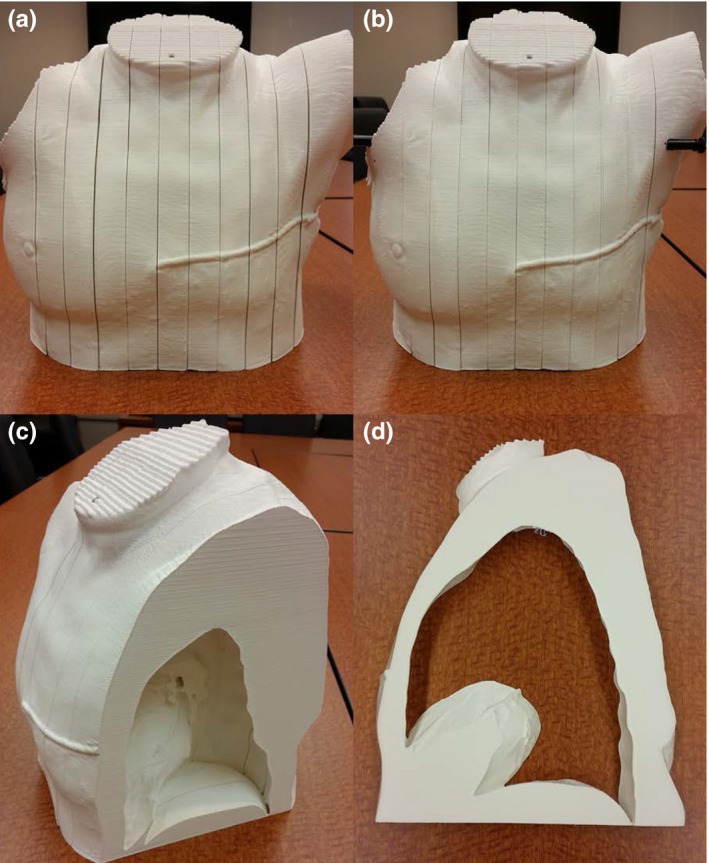
Photographs of the 3D‐printed phantom. (a) and (b) show the entire phantom before and after placement of immobilization rods, respectively. Note that gaps between slices were significantly reduced with the application of the immobilization rods. (c) View of the phantom with the two left‐most slices removed and (d) an individual slice.

Each slice printed correctly on its first attempt with the same settings. Pictures of the printed phantom before and after the immobilization rods were placed are shown in Fig. 3. The slices fit together well, and a high degree of detail was preserved.

The time to print each slice, the mass of each slice, and the cost of each slice are reported in Table 1. In total, the phantom took 267.5 hr to print, weighed 12.53 kg, and cost $524 in raw materials.

**Table 1:** Print statistics for each individual slice, average, and total

Slice	Print time (hr:min)	Mass (g)	Cost (US dollars)
1	31:41	1520.85	63.57
2	19:24	899.63	37.60
3	16:42	767.35	32.08
4	19:19	884.90	36.99
5	30:56	1446.55	60.47
6	35:14	1666.45	69.66
7	27:23	1267.11	52.97
8	19:48	913.50	38.18
9	17:28	801.73	33.51
10	22:28	1061.45	44.37
11	27:07	1296.15	54.18
Average	24:19	1138.70	47.60
Total	267:30	12,525.67	523.58

### 3.C Material analysis

We found that the printed PLA blocks had a mean HU of 160 ± 12, a physical density of 1.20 relative to water, and print errors of 1.09 mm on average.

**Figure 4:**
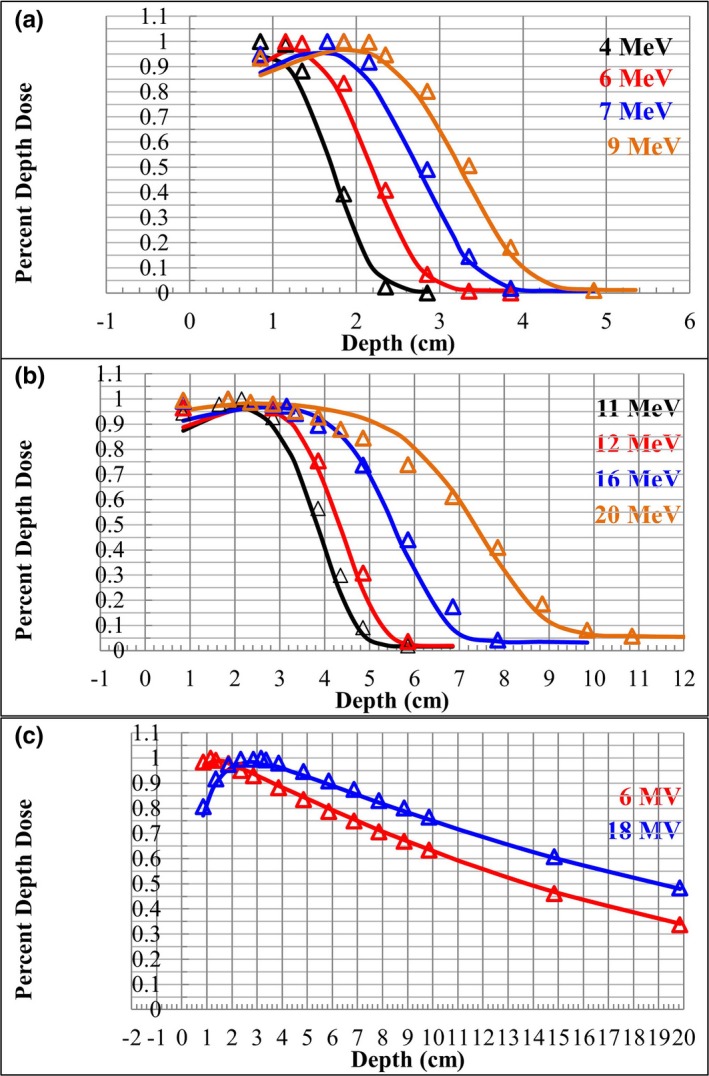
PDD curves measured (triangles) and calculated (solid lines) in PLA are shown. (a) Low‐energy electrons (4, 6, 7, and 9 MeV), (b) high‐energy electrons (11, 12, 16, and 20 MeV), and (c) 6‐ and 18‐MV photons.

The treatment planning system calculated PDDs and the measured PDDs are shown as solid lines and open triangles, respectively, in Fig. 4. The measured and calculated curves agreed within 2 mm for all electron energies other than 20 MeV, where differences up to 1 cm were observed. The 6‐ and 18‐MV photon beams measurements and calculations agreed within 2%.

### 3.D Phantom verification

**Figure 5:**
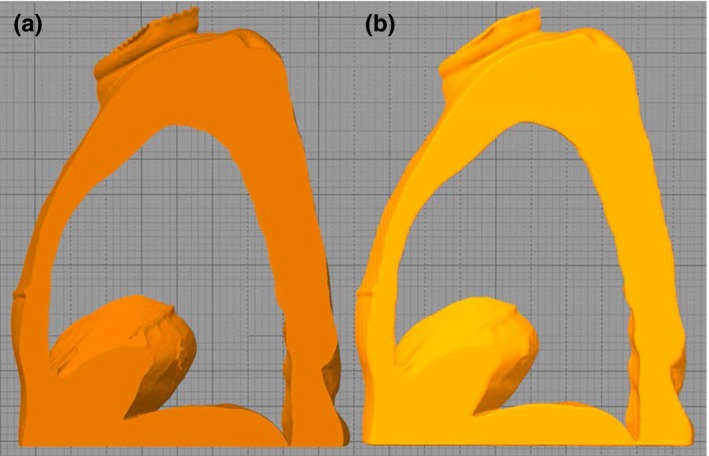
3D‐rendered models of slice number 8. (a) The planning model used to print slice 8, and (b) the model based on the CT scan of the actual printed slice. Figure 3(d) is a photograph of this same slice.

The measured slice errors were all positive, meaning the slices were consistently larger than planned, and ranged from 0.44 to 0.60 mm, with an average of 0.52 mm across all eleven slices. The average error at the bottom of the slices was 0.76 mm, while the errors at the middle and top were 0.51 and 0.29 mm, respectively. The decreasing error as the slice gets further from the print bed is consistent with previous observations that more warping occurs at the contact point between the printing surface and the object being printed. The volumetric errors of the individual slices ranged from 0.75% to 1.83%, with an average across all slices of 1.37%. The slices printed relatively homogenously and had a mean HU of 155 with a standard deviation of 18. Figure 5 shows an image of one slice as it was planned and as it was imaged.

**Figure 6:**
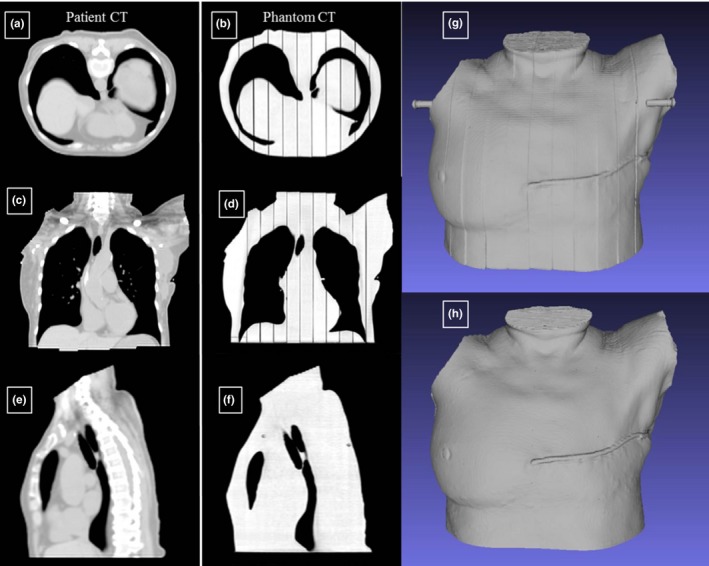
Comparison of the original patient CT scan registered with the completed phantom CT scan. Panels on the left show slices of the original patient CT scan, panels in the middle are from the CT scan of the assembled phantom, and panels on the right are the 3D‐rendered models of the original patient and the phantom. (a) and (b) Compare axial slices, (c) and (d) compare coronal slices, and (e) and (f) compare sagittal slices. (g) and (h) Compare rendered models of the phantom and patient CT scans, respectively.

There was excellent agreement between the original patient CT scan and the assembled phantom CT scan. Figure 6 shows slices of each CT scan next to each other, as well as the 3D model of the imaged phantom and original patient. The only disagreement between the phantom and patient data sets was in the lungs, where unsupported nodules were cropped to make printing possible (see Section 2.A).

The average CT numbers of the patient and printed phantom are reported in Table 2. In general, the printed phantom has a CT number around 140 HU higher than the patient's soft tissue, but is 100 HU lower than the patient's spinal area. The lungs of the phantom were also 130 HU lower than in the patient.

**Table 2:** CT number measurements for patient and phantom images

Location	Phantom CT number (HU)	Patient CT number (HU)	CT number discrepancy (HU)
Heart	133 ± 77	25 ± 25	108
Breast	82 ± 145	−61 ± 47	143
Arm	133 ± 90	−5 ± 95	138
Left lung	−989 ± 8	−862 ± 90	−128
Right lung	−993 ± 7	−861 ± 95	−132
Spine	132 ± 65	227 ± 164	−95

## 4 DISCUSSION

In this study, we successfully produced a patient‐specific, 3D printed phantom using a commercial printer and readily available software and materials. All software used in the design and preparation of print files was acquired or licensed for under $1000. The phantom was printed in eleven sagittal sections to minimize the impact of material warping. Although the overall phantom was large (35 cm × 25 cm × 32 cm), it had minimal warping (<2%). Because the small amount of warping observed was primarily in the inferior portion of the phantom (closest to the print bed during printing), the individual slices were well aligned in the fully assembled phantom. Accurate, simple, and inexpensive phantoms like these could be valuable for a variety of radiotherapy applications in both research and clinical settings. This particular phantom has been useful to us as a tool in developing breast compensators that can fit on irregular patient anatomy. Standard anthropomorphic phantoms are not designed to mimic patient treatment positioning, e.g., breast patients are simulated with their ipsilateral arm raised above the head. This type of patient‐specific phantom can be highly useful and informative in scenarios such as our present work designing patient‐specific compensators for breast cancer and many other treatment sites or patient‐specific QA of treatments where patients are in atypical treatment positions.

Our printed phantom differs from those described in other published work in several ways. Because of material warping, most 3D‐printed phantoms in the literature have been printed as a shell and then filled with liquid to make them solid.^1^, ^3^, ^4^ Our phantom was printed at 100% infill, which reduces the amount of work involved in the overall fabrication process. Concerns about material warping have also limited the size of most phantoms, as has the scarcity of large‐scale 3D printers. By printing slices in the sagittal orientation on the recently available Gigabot 2.0, we were able to create large (up to 2.5 cm × 23 cm × 32 cm) solid parts while still keeping warping errors under 2%.

One of the greatest advantages of our printed phantom is its relative cost. Including software, hardware, and materials, our phantom fabrication cost less than $15,000 and additional phantoms will cost only approximately $500 in materials. Traditionally manufactured standard anthropomorphic phantoms can cost more than $20,000, while patient‐specific ones cost much more. Related to cost is the simplicity of our process. The fabrication process and steps outlined in this study can be used on demand to rapidly design and produce not only full‐sized phantoms but any patient‐specific model of any size.

The limitation of the phantom is that both soft tissue and bone were represented by PLA. While the radiological properties of PLA are between those of bone and water, PLA is not a perfect approximation for either. The addition of a second material to simulate bone and give the phantom some heterogeneity would be advantageous. Some groups have reported work on variable‐density 3D‐printed phantoms, and while their work is promising, the variable density either has a high dependence on the direction of radiation^11^ or requires a complicated custom extrusion system and labor‐intensive postprocessing work.^2^ Recently, we acquired a new Gigabot 3D printer with an additional extruder with the capacity to print with multiple materials. Future work will include the addition of multiple‐material printing to the workflow described here.

It is an important point that PLA is not dosimetrically identical to water. Further, complex‐printed objects tend to have a lower HU than simple blocks, with an electron compensator having 107 HU compared to its 130 HU block,^10^ and our own phantom having a mean HU of 133, compared to our 160 HU blocks. In addition, PLA does not fall on a standard CT calibration curve due to its density of 1.20 relative to water. In order to accurately calculate dose in PLA, the HU or physical density must be manually overwritten. Burleson et al. found they could accurately calculate PDDs in PLA if they set the HU to 260,^5^ and in our own PLA PDD measurements, we set the density to 1.20 in order to get accurate calculations. In summary, the physical and radiological properties of PLA have been characterized and we have demonstrated that it can be used to fabricate patient‐specific single material radiation therapy phantoms. While implementation of multi‐material 3D printed phantoms would be a further improvement, homogeneous phantoms are at present routinely used for many different types of radiation treatment QA. Examples include patient‐specific IMRT QA and accreditation procedures for clinical trials.^15^, ^16^

Another limitation was the time required to print the phantom. While preparation was straightforward and not very time‐consuming, printing the entire phantom took over 11 days and positioning rods had to be manually drilled. In future iterations of phantoms, the positioning rod holes can be designed into the original slices, reducing the need for postprinting manual labor. Because of this long print time, large patient‐specific phantoms are not currently feasible for routine QA procedures done for every patient. With a well‐defined workflow, however, in‐house fabricated 3D‐printed phantoms can be an inexpensive, simple, and accurate alternative to commercially available phantoms and can be used to perform end‐to‐end QA of new radiation therapy techniques and for various research applications.

## 5 CONCLUSION

This study has shown that large patient‐specific phantoms can be fabricated with high accuracy on inexpensive, commercially available 3D printers. We successfully used real patient data to make an anatomically accurate, full‐scale phantom, overcoming previously encountered 3D printing limitations of size and material warping. Our method required no customized software or materials and was simple to execute. With multiple‐material extrusion printers, patient‐specific phantoms could be improved further and could soon be a viable tool for many research and clinical tasks.

## CONFLICTS OF INTEREST

The authors declare no conflict of interest.
